# Neural Stem Cell Tumorigenicity and Biodistribution Assessment for Phase I Clinical Trial in Parkinson’s Disease

**DOI:** 10.1038/srep34478

**Published:** 2016-09-30

**Authors:** Ibon Garitaonandia, Rodolfo Gonzalez, Trudy Christiansen-Weber, Tatiana Abramihina, Maxim Poustovoitov, Alexander Noskov, Glenn Sherman, Andrey Semechkin, Evan Snyder, Russell Kern

**Affiliations:** 1 International Stem Cell Corporation, Carlsbad, CA, USA; 2 Sanford-Burnham-Prebys Medical Discovery Institute, La Jolla, CA, USA

## Abstract

Human pluripotent stem cells (PSC) have the potential to revolutionize regenerative medicine. However undifferentiated PSC can form tumors and strict quality control measures and safety studies must be conducted before clinical translation. Here we describe preclinical tumorigenicity and biodistribution safety studies that were required by the US Food and Drug Administration (FDA) and Australian Therapeutic Goods Administration (TGA) prior to conducting a Phase I clinical trial evaluating the safety and tolerability of human parthenogenetic stem cell derived neural stem cells ISC-hpNSC for treating Parkinson’s disease (ClinicalTrials.gov Identifier NCT02452723). To mitigate the risk of having residual PSC in the final ISC-hpNSC population, we conducted sensitive *in vitro* assays using flow cytometry and qRT-PCR analyses and *in vivo* assays to determine acute toxicity, tumorigenicity and biodistribution. The results from these safety studies show the lack of residual undifferentiated PSC, negligible tumorigenic potential by ISC-hpNSC and provide additional assurance to their clinical application.

Human pluripotent stem cells (PSC), such as embryonic stem cells (hESC), induced pluripotent stem cells (iPSC), nuclear transfer embryonic stem cells (NT-ESC) and parthenogenetic stem cells (hpSC), have the capacity to self-renew and differentiate into any cell type in the body^1^,^2^,^3^,^4^. PSC differentiated derivatives are a promising source for cell based therapies because of their wide variety of treatment applications^5^. However, there are significant obstacles preventing their clinical translation due to the potential to form tumors by residual undifferentiated PSC^6^. Therefore, strict quality control measures and preclinical studies must be conducted to mitigate these risks and enable regulatory approval of a clinical trial^7^,^8^,^9^,^10^,^11^,^12^,^13^,^14^,^15^. After years of exhaustive preclinical development, a few PSC based trials have received approval for indications such as spinal cord injury, Stargardt’s disease, age-related macular degeneration, Type 1 diabetes, and Parkinson’s disease (PD)^16^,^17^,^18^,^19^,^20^,^21^,^22^,^23^,^24^,^25^. Interim results of these studies show that PSC based therapies are safe and well tolerated with no serious adverse events or teratomas^26^.

Here, we describe preclinical safety studies conducted in support of the approval of the first PSC-based therapy for Parkinson’s disease. This is a single arm, open-label, dose-escalating, Phase I study evaluating the safety and tolerability of human parthenogenetic derived neural stem cells ISC-hpNSC injected into the striatum and substantia nigra of patients with Parkinson’s disease (ClinicalTrials.gov Identifier NCT02452723)^20^,^27^,^28^. Parkinson’s disease (PD) is the second most common neurodegenerative disease affecting millions of people worldwide with primary motor signs including tremor, bradykinesia, rigidity and postural instability. There is currently no cure for PD and the available treatment options including pharmacological approaches and deep brain stimulation treat the symptoms but do not stop disease progression. PD is characterized by a loss of dopaminergic neurons in the substantia nigra and cell based therapies have the potential to protect and restore the nigrostriatal system. In this trial we use neural stem cells derived from hpSC for three main reasons. First, hpSC are derived from the chemical activation of unfertilized oocytes^3^,^29^, bypassing the ethical concerns associated with hESC because no human embryo is destroyed in their derivation^30^,^31^. Second, hpSC can be derived homozygous at the HLA loci from both heterozygous and homozygous donors^29^ with the potential to immune-match millions of patients if the HLA type is common^32^. A recent study comparing the frequencies of coding mutations in PSC has also shown that hpSC have lower number of *de novo* coding mutations than iPSC and NT-ESC^33^. Third, it was demonstrated that injecting clinical grade ISC-hpNSC into the striatum and substantia nigra promotes recovery by increasing dopaminergic cell number, innervation and dopamine levels in a non-human primate model of PD^28^. We believe ISC-hpNSC promote recovery by providing neurotrophic support and replacing lost dopaminergic neurons. This new therapeutic approach has the potential to slow down disease progression. ISC-hpNSC has other regenerative medicine applications such as traumatic brain injury, stroke and spinal cord regeneration. ISC-hpNSC could restore the neural circuits involved in central pattern generation of the injured spinal cord^34^.

ISC-hpNSC were derived through a chemically directed differentiation method^20^,^35^, expanded and cryopreserved into master and working cell banks under current good manufacturing practice (cGMP) conditions. In order to qualify these banks for clinical use, we implemented strict quality control measures to test for sterility, purity, identity and safety. One of the most important quality control measures is determining if there are any residual undifferentiated pluripotent hpSC in the final ISC-hpNSC population^8^. To evaluate this safety concern, a panel of *in vitro* assays was performed, which included culturing ISC-hpNSC in media that favor hpSC growth, flow cytometry and qRT-PCR analysis to detect residual undifferentiated hpSC based on the US FDA’s feedback. To study the tumorigenic potential of ISC-hpNSC, two *in vivo* safety studies were performed: an acute toxicity study and a 9-month tumorigenicity and biodistribution study with escalating doses of ISC-hpNSC in immunodeficient athymic nude rats. The results from these safety studies demonstrate that the residual number of undifferentiated hpSC is negligible and the tumorigenic potential of ISC-hpNSC by either malignant transformation or uncontrolled growth is insignificant.

## Results

### cGMP manufacturing, characterization and testing of ISC-hpNSC

**Figure 1:**
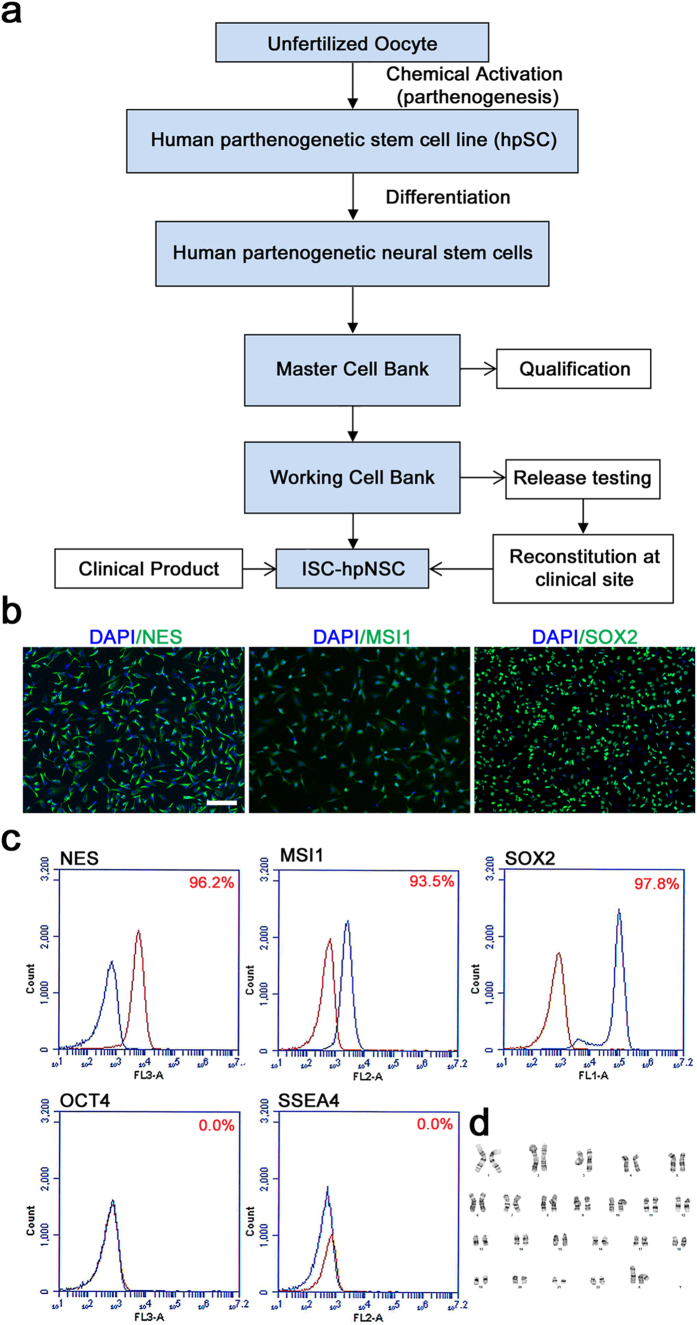
Characterization of ISC-hpNSC manufactured under cGMP. (**a**) Derivation and cGMP manufacturing scheme of ISC-hpNSC. (**b**) Immunocytochemical analysis of ISC-hpNSC for neural markers Nestin (NES), Musashi (MSI1) and SOX2. (**c**) Quantitation by flow cytometry analysis of neural markers NES, MSI1, and SOX2 and pluripotency markers OCT-4 and SSEA-4. Percentage of positive cells (blue) is calculated based on isotype control stained cells (red). (**d**) G-banding karyotyping analysis shows that the cells have a normal female 46 XX karyotype.

The derivation process of ISC-hpNSC began with the parthenogenetic activation of an unfertilized oocyte from a consented donor, followed by cultivation of the resulting blastocyst, and subsequent selection and establishment of the human parthenogenetic stem cell (hpSC) line (LLC2P)^3^ (Fig. 1a). The hpSC line was chemically directed to differentiate into a homogenous population of neural stem cells (ISC-hpNSC) under feeder-free and cGMP conditions. ISC-hpNSC were then expanded and cryopreserved to generate master and working cell banks that were qualified and underwent testing before release to the clinical site (Fig. 1a). ISC-hpNSC were negative for bacterial, fungal, mycoplasma and adventitious viral contaminants as determined by a combination of *in vitro* and *in vivo* assays, quantitative PCR/RT-PCR, and thin section transmission electron microscopy (Table 1). ISC-hpNSC had a 46XX normal karyotype, expressed neural markers and no pluripotency markers (Fig. 1b,c,d). Short tandem repeat analysis and HLA typing confirmed that ISC-hpNSC had an identical DNA fingerprint match and HLA type as the parent hpSC cell line (LLC2P)^3^.

### *In Vitro* culture experiment to detect residual hpSC

The residual presence of undifferentiated hpSC is a safety concern due to their potential to form tumors. We attempted to detect residual undifferentiated hpSC in our clinical grade ISC-hpNSC population by *in vitro* culture experiments. In these experiments, we altered the tissue culture conditions of ISC-hpNSC such that they would be favorable to hpSC growth. Under favorable hpSC culture conditions, rare surviving hpSC in ISC-hpNSC cultures would become more numerous and therefore easier to detect. Control wells, consisting of spiked hpSC in ISC-hpNSC cultures, provided confirmation that residual hpSC can in fact grow under the altered *in vitro* culture conditions. The ISC-hpNSC were grown either as a pure population (100% ISC-hpNSC) or spiked with 0.1, 0.01 or 0.001% hpSC in Essential 8 medium, which favors the growth of hpSC. As separate controls, pure (100%) hpSC were grown in conditions that favor hpSC (Essential 8 medium) and conditions that favor ISC-hpNSC (StemPro NSC medium) growth. All the conditions were seeded with the same number of cells and after one week of culture, colonies of hpSC were observed in the spiked 0.1% and 0.01% hpSC culture conditions and none in the 0.001% spiked condition (Table 2). The presence of the hpSC colonies was confirmed by flow cytometry analysis of OCT-4 expression. The 0.001% hpSC spiked condition had no colonies but a small detectable OCT-4 signal. However, no hpSC colonies or OCT-4 expression were detected in the pure, unspiked ISC-hpNSC culture (Table 2). Pure (100%) hpSC reached confluence within four days in their optimal medium (Essential 8 medium) and managed to survive under ISC-hpNSC proliferative conditions (StemPro NSC medium), but at vastly reduced numbers indicating that the ISC-hpNSC culture conditions are not permissive for hpSC growth (Table 2). These results indicate that hpSC colonies are detectable under permissive conditions within a population of ISC-hpNSC if they are present in numbers equal to or exceeding 0.01%. The lack of hpSC colonies or OCT-4 expression in the pure, unspiked ISC-hpNSC culture indicates that their hpSC content is below 0.001%.

### Magnetic separation and flow cytometry analysis to detect residual hpSC

**Figure 2:**
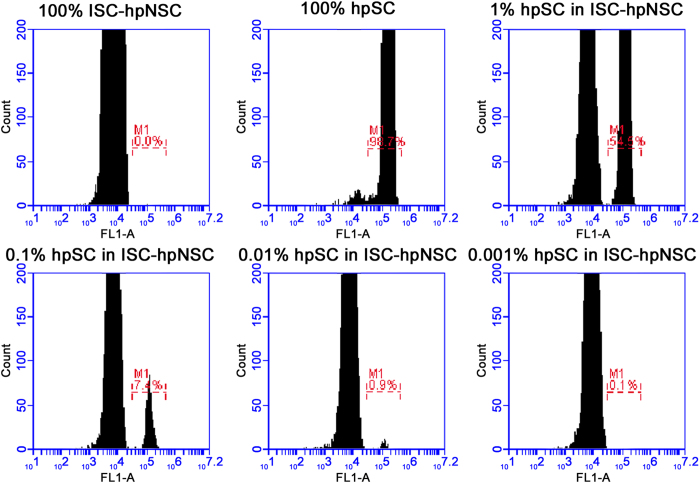
Flow cytometry analysis after magnetic separation of ISC-hpNSC and hpSC populations. Flow cytometry analysis of OCT-4 expression of the different cell populations in this experiment: 100% ISC-hpNSC, 100% hpSC, 1% hpSC in ISC-hpNSC, 0.1% hpSC in ISC-hpNSC, 0.01% hpSC in ISC-hpNSC, and 0.001% hpSC in ISC-hpNSC population. The pure 100% ISC-hpNSC was the only cell population without OCT-4 expression signal.

Rare cell populations are difficult to detect and one solution to this problem is to employ debulking methods, such as cell separation with magnetic columns. Scarce or rare hpSC can be purified from the ISC-hpNSC population by magnetic column purification to increase the percentage of any residual hpSC population that might be present. We used anti-TRA-1-60 microbeads for positive selection purification of hpSC followed by flow cytometry analysis for OCT-4 expression. Control hpSC were spiked into ISC-hpNSC populations as 1%, 0.1%, 0.01% and 0.001% in an effort to demonstrate that if residual hpSC were in fact present, they would be detectable once the major population of ISC-hpNSC had been debulked by magnetic purification. Cell populations of 100% hpSC and 100% ISC-hpNSC were used as controls for the experiment and to set up gates specific to the population sizes and exclude debris. Controls for the flow cytometry analysis were determined with isotype control stains of the pure ISC-hpNSC and hpSC populations. Flow cytometry analysis demonstrated that there was a clear hpSC peak of OCT-4 positive cells as low as 0.01% spiked-in hpSC (Fig. 2). No hpSC peak was detected in the 0.001% spiked-in hpSC population but a few flow cytometry events were detected with OCT-4 signal (Fig. 2). No hpSC peaks or OCT-4 positive events were detected in the pure ISC-hpNSC population demonstrating that unspiked ISC-hpNSC populations could potentially have ≤0.001% residual hpSC (Fig. 2).

### qRT-PCR assay to detect residual hpSC

**Figure 3:**
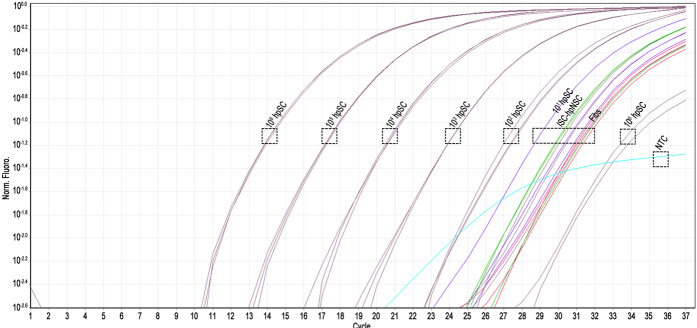
qRT-PCR analysis of ISC-hpNSC. Log curves for quantitation of the qRT-PCR analysis of ISC-hpNSC with dotted squares indicating the groupings of each sample run in triplicate. ISC-hpNSC and human fibroblasts group with the same level of *POU5F1* expression of 10 hpSC. The negative control (NC) samples are below the 1 hpSC (10^0^ hpSC) cell reaction. Linearity of curves Ct was between 17–35 cycles.

The third *in vitro* method used for detecting residual hpSC in the clinical grade ISC-hpNSC was a qRT-PCR assay. We attempted to detect the lowest hpSC levels by analyzing the expression of the pluripotency gene *POU5F1*, which encodes the OCT-4 protein. RNA from hpSC, ISC-hpNSC and human fibroblasts was purified and reverse transcribed. Human fibroblasts were included as a negative control because they are the least likely cell population to harbor residual *POU5F1* expression. hpSC cDNA was diluted in 10-fold increments from the equivalent of 10^6^ cells to 0.1 cell. Samples were subjected to QPCR and expression of *POU5F1* was directly compared to expression in hpSC. Furthermore, additional controls consisted of water substituting for template during QPCR and omission of reverse transcriptase to establish that genomic DNA was not being amplified. Melting curves were also utilized to verify the creation of reaction-specific PCR product. The melting curves demonstrated that the quantified read-out was the result of a specific product and not an artifact and specific *POU5F1* product was present in all samples except the water-only control and the reverse transcriptase negative controls. The reverse transcriptase negative controls also demonstrated that the signal we observe is not due to amplification of genomic DNA. With a completely pure hpSC sample, we are able to detect as little as one hpSC cell equivalent from a dilution curve beginning at 10^6^ hpSC, which corresponds to 0.0001% hpSC (Fig. 3). When expression of *POU5F1* in this dilution curve is compared against expression of *POU5F1* in human fibroblasts or ISC-hpNSC, we can estimate roughly 10 cells of human fibroblasts or ISC-hpNSC out of 10^6^ or 0.001% are expressing *POU5F1* (Fig. 3). The potential presence of slightly over 1 hpSC cell in 100,000 ISC-hpNSC, or 0.001%, is very similar to the previously reported limit of detection by qRT-PCR^8^. Since, human fibroblast cultures do not harbor pluripotent *POU5F1* positive cell populations, it is likely that the observed levels of *POU5F1* expression found in both ISC-hpNSC and in fibroblast cultures represent normal background levels found in both cultures rather than percentages of pluripotent cells. Human fibroblasts are very well known for their inability to immortalize or become tumorigenic unless they receive multiple genetic insults or undergo strenuous reprogramming efforts to induce pluripotency^2^. The residual expression of *POU5F1* in human fibroblasts and ISC-hpNSC may be taken as a measure of a safe threshold of *POU5F1* expression in ISC-hpNSC. These three different *in vitro* measurements of hpSC in our clinical grade ISC-hpNSC population agree that the residual hpSC population is less than 0.01% with our final test indicating roughly 1 in 100,000 cells (0.001%), equal to levels found in human fibroblasts.

### Acute toxicity study of ISC-hpNSC

The acute toxicity of ISC-hpNSC was determined in immunodeficient athymic nude rats. We chose immunodeficient athymic nude rat model to minimize the potential of xenograft rejection and accommodate the clinically relevant doses of ISC-hpNSC in the brain that would not be possible in smaller immunodeficient mouse models. ISC-hpNSC were transplanted by stereotactic injection into the striatum and substantia nigra to mimic the clinical route, as recommended by the US FDA for the design of preclinical studies^11^. Nonclinical routes of administration do not adequately account for the influence of the local host microenvironment, which could affect the product’s ability to form tumors^11^. The acute toxicity studies were used to set an appropriate dose level in animals, give an early indication of target organ toxicity and support the effects of overdose in humans^36^. Escalating doses of ISC-hpNSC were tested to determine the maximum feasible dose (MFD) of ISC-hpNSC, which is the dose that can be safely injected without adverse impact to the cells or the animals due to surgical delivery procedure. The MFD is limited by the maximum concentration that can be achieved as a cell suspension and the maximum dose volume that can practically be administered to the animal^37^. In this study, male and female rats received vehicle control and three escalating doses of ISC-hpNSC by increasing the cell concentration and keeping the total injection volume constant at 36 μl, which is the maximum volume that can be safely injected into striatum and substantia nigra of the rat^38^,^39^,^40^ (Supplementary Table S1). The cell concentrations and doses tested were approximately 70,000 cells/μl, 153,000 cells/μl, and 214,000 cells/μl and 2.5 × 10^6^ cells, 5.5 × 10^6^ cells, and 7.7 × 10^6^ cells, respectively (Supplementary Table S1). The highest dose of 7.7 million cells in rats is equivalent to over 2.2 billion cells in humans, based on the inter-species volume comparison of the striatum and substantia nigra^38^,^40^,^41^,^42^. The MFD of 2.2 billion cells far exceeds, by more than 30 fold, the doses administered in the Phase 1 trial, providing a high safety margin.

**Figure 4:**
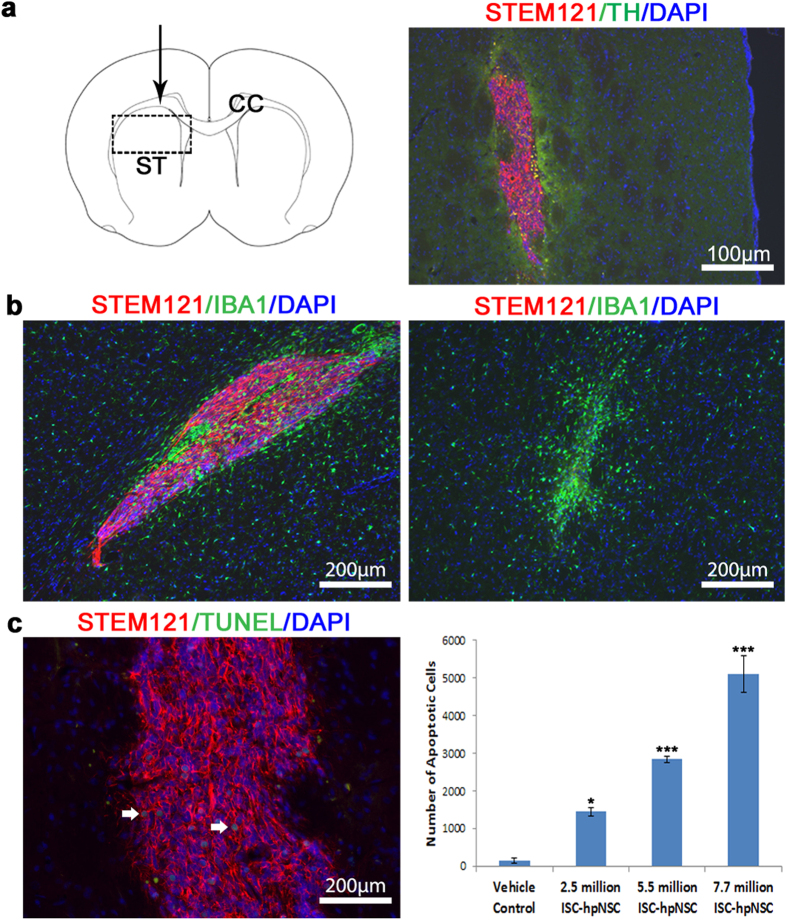
Acute toxicity study of ISC-hpNSC. (**a**) Engraftment of ISC-hpNSC in the striatum of athymic nude rats. On the left is the coronal section with the dotted rectangle showing the location where the ISC-hpNSC graft was found. The black arrow represents the direction of the injection site. ST: striatum; CC: corpus callosum. On the right is a higher magnification micrograph showing the ISC-hpNSC graft stained for anti-STEM121 (red), anti-tyrosine hydroxylase (green), and DAPI (blue). (**b**) IBA-1+ staining comparison between rats receiving ISC-hpNSC or vehicle control. On the left is the ISC-hpNSC graft and on the right is the injection site in the vehicle control animal stained for anti-STEM121 (red), anti-IBA-1 (green) and DAPI. The number of IBA-1+ microglia cells surrounding the ISC-hpNSC graft is comparable to the number found at the injection site in the vehicle control animals. (**c**) TUNEL staining and quantification of apoptotic cells in animals injected with vehicle control and escalating doses of ISC-hpNSC. White arrows point to the apoptotic cells. Data was expressed as average ± SEM, one-factor ANOVA with Dunnett test comparing number of apoptotic cells of ISC-hpNSC doses against vehicle control: n = 3; α = 0.05; ***P < 0.001; *P < 0.05.

The safety of the intranigrostriatal administration of ISC-hpNSC was assessed by clinical observations, body weights, necropsy, histopathological and immunohistochemical analysis. In-cage clinical observations and physical examinations determined that the rodents safely tolerated the high ISC-hpNSC doses with no adverse events. There were no statistical statistically significant differences in body weight changes between treated and vehicle control animals. Seven days post-transplantation, the animals were sacrificed and perfused and the brains were collected for analysis. Gross necropsy did not reveal any abnormalities, tumors or ectopic tissue formation and histopathological analysis by board-certified veterinary pathologist did not detect the presence of tumors (Fig. 4a). Immunohistochemical analysis confirmed engraftment of ISC-hpNSC at all three doses and low signs of immune rejection, with similar numbers of IBA-1^+^ cells around the injection site to the vehicle control injected animals (Fig. 4b). The reduction of the host immune response and immunomodulatory properties of neural stem cells by the secretion of cytokines such as TGF-β1 and TGF-β2 is a well-documented fact^43^,^44^. As expected, there were more apoptotic cells in the ISC-hpNSC transplanted than the vehicle control animals, with the number increasing with cell dose (Fig. 4c). Based on the results of this study, it was determined that the maximum feasible dose of ISC-hpNSC that can be safely administered in the rat brain is 7.7 million cells, equivalent to 2.2 billion cells in humans.

### Tumorigenicity and biodistribution study of ISC-hpNSC

**Figure 5:**
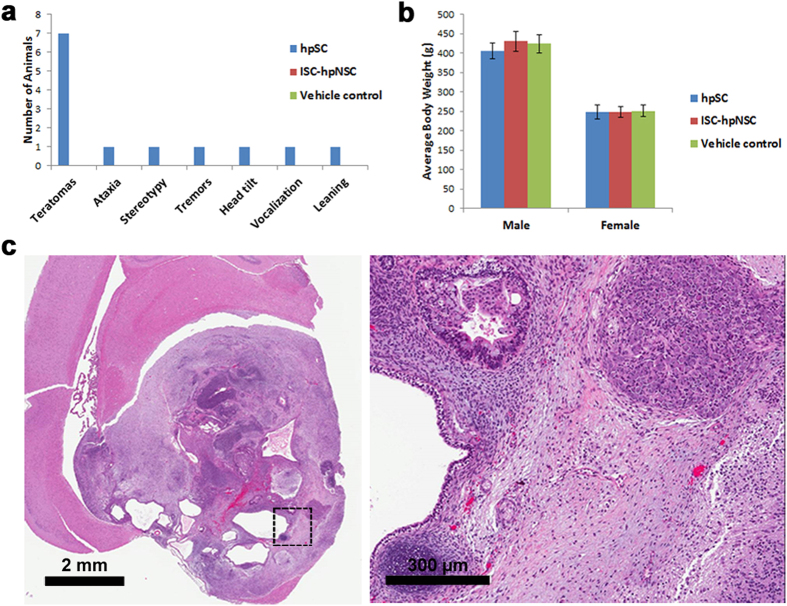
Tumorigenicity and biodistribution study of ISC-hpNSC. (**a**) Number of animals with teratomas, ataxia, stereotypy, tremors, head tilt, vocalization, and leaning in the hpSC, ISC-hpNSC, and vehicle control groups. (**b**) Average body weight of the hpSC, ISC-hpNSC, and vehicle control groups with no statistically significant differences between the groups. (**c**) Representative image of a teratoma found in the brain of a rat injected with undifferentiated hpSC with disorderly growth with red blood cells (red area) and numerous clear spaces. The black dotted square represents the area where the higher magnification image on the right was taken, showing the presence of cartilage (bottom left corner), mucous-producing cells (top left) and nervous tissue, representing disorderly endodermal and ectodermal differentiation as assessed by an experienced board certified pathologist.

Tumorigenicity testing allows the highly sensitive detection of both residual undifferentiated pluripotent hpSC and tumorigenic transformed cells in the final ISC-hpNSC population^12^. Therefore the results of the tumorigenicity tests are critical to assess the safety before human application^45^. The MFD dose determined in the acute toxicity study was evaluated in a GLP-compliant 9-month tumorigenicity and biodistribution study along with vehicle control, hpSC, and two doses of ISC-hpNSC that bracket the intended clinical dose, in 300 athymic nude rats (Supplementary Table S2). A group receiving undifferentiated hpSC was included as a positive control because hpSC are pluripotent cells that can form tumors within 8 weeks of implantation^3^,^29^. Both male and female rats received bilateral injections in the striatum and substantia nigra. Tumorigenicity testing was performed via the clinical route of administration to recapitulate the fate of transplanted cells in a microenvironment of host tissue. Safety was evaluated through clinical observations, food consumption, body weight recording, clinical pathology, necropsy, tumorigenicity analysis, histopathology, and biodistribution analysis. No ISC-hpNSC related adverse clinical observations were observed, whereas ataxia, stereotypy, tremors and weight loss were observed in some of the animals receiving hpSC, which resulted in *in extremis* euthanasia (Fig. 5a). Body weight changes were within expected biological variability and no test article related changes were noted in body weights in any group (Fig. 5b). Food consumption values were within expected biological variability and no test article-related changes were noted in any group. Intracranial administration of ISC-hpNSC to nude rats did not result in any meaningful effects on hematology or clinical chemistry endpoints at any collection interval. Hematology and clinical chemistry measures were within normal limits for this species and there were no statistical significant differences between vehicle control and treated samples. No test article-related deaths were observed in any of the animals injected with ISC-hpNSC, even those injected with the MFD. On the other hand, there were test article-related deaths in five animals injected with hpSC (two males and three females). Relevant macroscopic findings detected in as early as 7–8 weeks were a mass in the brain of these animals, which correlated microscopically to teratomas, with fully differentiated tissue that did not metastasize (Fig. 5a). These findings demonstrate that this model is sensitive to intracranial tumor formation following administration of undifferentiated hpSC. Teratomas consisted of two or more well-differentiated lines from neuroectoderm, mesoderm, and endoderm and occasionally compressed and/or caused tissue loss in the affected brains (Fig. 5c). Ciliated tall columnar epithelial cells and cartilage were often seen in teratomas. Postmortem, macroscopic and microscopic histopathological analysis by board-certified veterinary pathologist did not detect the presence of teratomas or tumorigenic risk in any of the animals injected with ISC-hpNSC. Additionally, injection of ISC-hpNSC cells was not associated with any proliferative risk or other serious adverse event at any dose or time interval. Biodistribution analysis by immunohistochemistry and qPCR did not detect ISC-hpNSC in peripheral organs, further supporting their safety profile.

## Discussion

PSC differentiated derivatives have the potential to treat countless unmet medical needs. However, in order for their clinical translation to become a reality, their safety and lack of tumorigenicity have to be demonstrated. In this study, we conducted a series of *in vitro* and *in vivo* preclinical studies to show that ISC-hpNSC are devoid of undifferentiated pluripotent hpSC and are safe for transplantation. The ISC-hpNSC were manufactured under cGMP conditions and tested for sterility, purity, identity and safety. The clinical grade ISC-hpNSC expressed neural markers and no pluripotency markers, had a normal karyotype and were negative for bacteria, fungal, mycoplasma and adventitious viral contaminants. Additionally, ISC-hpNSC were tested by *in vitro* and *in vivo* safety assays to determine that no residual undifferentiated pluripotent hpSC remained in the final cell preparation. In the first *in vitro* assay, ISC-hpNSC were cultured in medium that supports the growth of undifferentiated pluripotent hpSC along with spiked serial-fold dilutions of 0.1%, 0.01% and 0.001% hpSC. After a week in culture, hpSC colonies or OCT-4 expression were detected in the spiked cultures but not in the pure ISC-hpNSC population, indicating that ISC-hpNSC are either devoid of undifferentiated hpSC or their presence is below 0.001%. The second *in vitro* assay was a magnetic purification followed by flow cytometry analysis of pure ISC-hpNSC or spiked with 1%, 0.1%, 0.01% and 0.001% hpSC in an effort to demonstrate that if residual hpSC were in fact present, they would be detected once they had been concentrated by magnetic purification. The results of this assay show that an OCT-4 signal was still detected in the spiked 0.001% hpSC culture but not in the pure ISC-hpNSC population, providing additional supportive evidence that undifferentiated pluripotent hpSC are absent from ISC-hpNSC or present at concentrations lower than 0.001%. The third *in vitro* assay used to detect residual hpSC consisted of analyzing gene expression of *POU5F1* in ISC-hpNSC by qRT-PCR. The assay showed that ISC-hpNSC had the same level of *POU5F1* expression as the negative human fibroblast controls and it was equivalent to 0.001% hpSC. Based on the fact that human fibroblasts are known for their lack of *POU5F1* expression, this can be taken as a measure of a safe threshold for the lack of *POU5F1* expression in ISC-hpNSC.

In the *in vivo* assays, the acute toxicity study demonstrated that ISC-hpNSC can be tolerated at very high doses in the brain of immunodeficient rats. The MFD that can be safely administered bilaterally into the striatum and substantia nigra is 7.7 million ISC-hpNSC, which would be equivalent to administering 2.2 billion cells in humans. No abnormalities, ectopic tissue formation or the presence of tumors were detected. The ISC-hpNSC engrafted at all doses tested with low signs of immune rejection. As expected, we observed increased number of apoptotic cells increasing with the ISC-hpNSC dose, which could be explained by the host microglia rejecting excessive progenitor cells to maintain brain homeostasis^46^. The MFD established in the acute toxicity study was used in the second *in vivo* assay, a 9 month tumorigenicity and biodistribution GLP study evaluating bilateral administration of ISC-hpNSC into the striatum and substantia nigra. Testing the tumorigenicity of ISC-hpNSC via the clinical route of administration allows for the detection of not only rare cell populations of undifferentiated hpSC but also tumors formed by a spontaneous malignant transformation of the cells after transplantation into the nigrostriatal niche. In this study, no teratomas or tumorigenic risk were detected in any of the animals injected with ISC-hpNSC. On the other hand, teratomas were observed in some of the animals injected with the undifferentiated pluripotent hpSC in as early as 7–8 weeks, supporting the sensitivity of the model for detecting intracranial tumor formation during the time period evaluated. Injection of ISC-hpNSC did not cause any proliferative risk, serious adverse events or biodistribution of cells to peripheral organs.

Based on the extensive characterization and testing of the clinical grade ISC-hpNSC and the results of the *in vitro* and *in vivo* safety assays, with the number of animals used, the large doses administered, the duration of the monitoring period and the sensitivity to detect tumors in immunodeficient rodents via the clinical route of administration, we conclude that the tumorigenic potential of the ISC-hpNSC cells is negligible.

## Methods

### Testing and cGMP manufacturing of ISC-hpNSC

Human parthenogenetic stem cell (hpSC) line LLC2P (International Stem Cell Corporation, Carlsbad, CA, USA)^3^ was initially cultured under cGMP conditions in Knockout DMEM/F12 supplemented with 15% Knockout Serum Replacement, 2 mM GlutaMAX, 0.1 mM Minimum Essential Medium (MEM) nonessential amino acids, 0.1 mM β-mercaptoethanol and 5 ng/ml basic fibroblast growth factor (bFGF) (Thermo Fisher Scientific, Carlsbad, CA, USA) on human neonatal skin fibroblasts (International Stem Cell Corporation) with mechanical passaging to preserve the genetic integrity of hpSC^47^,^48^,^49^. The cells were then transferred to feeder-free and xeno-free conditions in Essential 8 Medium on Cell Therapy Systems (CTS) CELLstart substrate and subcultured with StemPro Accutase (all from Thermo Fisher Scientific) for three passages before neural induction. Then, when the culture reached 80% confluency, it was treated with 5 μM SB218078 and 1 μM DMH-1 (both from Tocris, Bristol, UK) in Knockout DMEM/F12, 1X GlutaMax, 1X N2/B27 Supplement (Thermo Fisher Scientific) for 11 days^20^,^35^. Neuralized hpSC were dissociated with StemPro Accutase and Y-27632 dihydrochloride (Tocris) and plated on CTS CELLstart-coated dishes in StemPro NSC Serum Free Medium (SFM) (Thermo Fisher Scientific) which consists of Knockout DMEM/F12 with StemPro Neural Supplement, 1X Glutamax, 20 ng/mL bFGF and 20 ng/mL epidermal growth factor (EGF). Human parthenogenetic derived neural stem cells (ISC-hpNSC) were expanded and cryopreserved to generate master and working cell banks. ISC-hpNSC were extensively tested for expression of neural and pluripotency markers, sterility, mycoplasma, karyotype, short tandem repeat analysis, *in vitro* assays for adventitious viral contaminants, *in vivo* assays for adventitious viruses, a comprehensive panel of human viruses by quantitative PCR/RT-PCR, ultrastructure cell morphology and viral detection by thin section transmission electron microscopy, and PCR-based reverse transcription assay for retrovirus detection. ISC-hpNSC is available upon request to research groups that desire to collaborate.

### Flow cytometry analysis

Control ISC-hpNSC, control hpSC and spiked samples were washed once with DPBS without Calcium or Magnesium DPBS^**−/−**^ (LifeLine Cell Technology, Oceanside, CA, USA) and resuspended in 4% paraformaldehyde in PBS (Affymetrix, Santa Clara, CA, USA) for 30 min at 4 °C. Samples were then washed twice in 1% BSA in DPBS^**−/−**^ and stored at 4 °C. The following day samples were permeabilized in 1% BSA/0.1% Triton X-100 (Sigma Aldrich, St. Louis, MO, USA) in DPBS^**−/−**^ at room temperature for 30 min. Samples were then labelled with antibodies for 1–2 hours at 4 °C. Samples were washed three times with 1% BSA/0.1% Triton X-100 in DPBS^**−/−**^ and subjected to flow cytometry on a BD Accuri C6 flow cytometer. Unstained controls were run in a forward scatter (FSC) vs side scatter (SSC) window to establish location of ISC-hpNSC and hpSC populations. Gates on each FSC vs SSC population stained with OCT-4 in a FL1 histograph established negative or positive populations. Spiked samples were then run and evaluated.

### Magnetic column separation

Cells in separation buffer, which consisted of DPBS^**−/−**^, 0.5% BSA and 2 mM EDTA (Sigma Aldrich) degassed and chilled to 4 °C, were either stained with anti-TRA-1-60 microbeads (Miltenyi Biotech, San Diego, CA, USA) as separate pure populations or after spiking ISC-hpNSC with hpSC. Cell populations were run over LS columns (Miltenyi Biotech) for positive selection of TRA-1-60 bearing cells as specified by manufacturer. Yields were then labeled with fluorescent antibodies for flow cytometry analysis.

### qRT-PCR

Cells were harvested with Accutase, washed once with DPBS^**−/−**^ counted and lysed in RLT buffer from the RNeasy Mini Plus Kit (Qiagen, Valencia, CA, USA) with β-mercaptoethanol (Sigma Aldrich). RNA was isolated and purified using the RNeasy Mini Plus Kit according to manufacturer’s instructions. RNA was reverse transcribed using iScript cDNA Synthesis Kit (BioRad Laboratories, Irvine, CA) according to manufacturer’s directions, using a PX2 Thermocycler (Thermo Fisher Scientific). Transcribed cDNA was amplified in triplicate via QPCR using Rotor-Gene Q cycler and the Rotor-Gene SYBR Green PCR Kit (Qiagen). Primers were Hs_POU5F1_1_SG (Cat #QT00210840) and Hs_PPIG_1_SG (Cyclophilin G, Cat #QT00084259) from Qiagen. Cyclophilin G was utilized to normalize variations between samples.

### Transplantation of ISC-hpNSC in the acute toxicity study

Immunodeficient male and female athymic nude rats (Csl:NIH-Foxn1rnu strain code 316 (homozygous) (Charles River Laboratories, San Diego, CA, USA) received bilateral injections of ISC-hpNSC with three injection tracks per hemisphere: two tracks for the striatum and one for the substantia nigra, totaling 6 injection tracks per brain (Supplementary Table S3). A 26 g 10 μL Hamilton syringe was lowered to the appropriate ventral coordinates with the tooth bar at −3.3 mm (Supplementary Table S3). In each hemisphere, four deposits of 2 μL each were made per track in the striatum, separated every 0.5 mm. A single deposit of 2 μl was made in each substantia nigra. Each cell deposit was 2 μL with a varying concentration of cells/μl (Supplementary Table S1) for a total volume of 36 μL per brain. The test article infusion rate was 1.0 μL/min. Following infusion, the syringe was left in place for 5 minutes to avoid back diffusion, and then slowly withdrawn. The incision was closed by sutures or wound clips. All procedures were done following the National Institutes of Health (NIH) Guide for the Care and Use of Laboratory Animals and were approved by the Institutional Animal Care and Use Committee (IACUC) of BTS Research (San Diego, CA, USA).

### Transplantation of ISC-hpNSC in the tumorigenicity and biodistribution study

Using a standard, by weight, measured value randomization procedure, 150 male and 150 female immunodeficient athymic nude rats (weighing 164 to 273 g and 120 to 218 g, respectively, prior to surgery) were assigned to the study (Supplementary Table 2). Surgery was conducted on rats that were 8 weeks ± 4 days of age. Animals were placed in a stereotaxic frame and a midline skin incision was made over the skull. The skin was retracted and bregma identified. Burr holes were drilled through the cranium at the desired coordinates to permit the introduction of a 26 gauge point style AS needle into the area of the striatum and substantia nigra for a total of four or six injection tracts per brain. Each tract consisted of one, two, or four injection sites. The coordinates used for male and female animals were the same as the acute toxicity study (Supplementary Table S4). The anterior/posterior and right/left values are relative to bregma and dorsal/ventral values are relative to the surface of the dura at the injection site. A qualified and calibrated digital stereotaxic instrument was used and therefore no final coordinate calculations were required. Coordinates were set to zero when bregma was reached and the depth was set to zero when the dura was reached. The vehicle DPBS, positive control hpSC, or ISC-hpNSC, were administered at 2 μL/injection site delivered at a rate of 1 μL/minute, for a total dose volume of 12 μL for all the animals instead of the ones receiving the maximum feasible dose of ISC-hpNSC which received a total dose volume of 36 μL (Supplementary Table S2). The needle was allowed to remain in place for 60 to 90 seconds after completion of dosing at each injection site. Following the injections, the skin was closed using standard procedures and tissue glue. All procedures were done following the NIH Guide for the Care and Use of Laboratory Animals and were approved by the IACUC of MPI Research (Mattawan, MI, USA).

### Immunohistochemistry

Brain sections were washed in Tris buffer (0.1 M Tris, 0.85% NaCl, pH 7.5) for 5 min. and incubated for 20 min. in 3% H_2_O_2_, 10% Methanol in Tris buffer (Sigma-Aldrich). The sections were then washed for 5 min. in Tris buffer, 15 min. in wash buffer (0.1% Triton X-100 in Tris buffer), and 15 min. in blocking buffer (2% BSA, 0.1% Triton X-100 in Tris buffer) (Sigma-Aldrich). Sections were incubated with primary antibody diluted in blocking buffer at room temperature overnight with shaking. Sections were washed for 15 min. in wash buffer and 15 min. in blocking buffer, and incubated for 1 hour at room temperature with secondary antibody diluted in blocking buffer. Sections were washed four times and coverslips were mounted on the slides with VECTASHIELD Hard Set Mounting Medium with 4,6-diamidino-2-phenylindole (DAPI) (Vector Laboratories, Burlingame, CA). Imaging was performed using an Axioimager. M2 ApoTome.2 microscope equipped with a AxioCam MRm camera (Zeiss, San Diego, CA, USA). Antibodies used are described in Supplementary Table S5.

### Terminal deoxynucleotidyl transferase dUTP nick end labeling (TUNEL)

TUNEL was performed to determine the extent of apoptosis in brains transplanted with ISC-hpNSC. Sections were permeabilized with 0.1% Triton-X-100, in 0.1% sodium citrate for 5 min at 4 °C. The sections were washed twice with DPBS and then incubated with a labeling solution consisting of terminal deoxynucleotidyl transferase and nucleotide mixture in a ratio of 1:10 obtained from the *In-Situ* Cell Death Detection Kit-Fluorescein (Roche Applied Science, Indianapolis, IN) at 37 °C for 2 h. Sections were then washed three times with DPBS and mounted using VECTASHIELD Hard Set Mounting Medium with DAPI (Vector Laboratories).

### Stereolgical analysis

Number of TUNEL positive cells in the striatal region graft site were estimated using an Axioimager.M2 ApoTome.2 microscope (Zeiss) and Stereo Investigator software (MBF Bioscience, Williston, VT, USA). Contours were drawn at 2.5 × objective outlining areas of human cell engraftment and counting was performed at 63 × magnification. The counting frame utilized was 50 × 50 μm with a grid size of 118.8 × 118.8 μm. The sampling grid was adjusted so that 20% of the selected region was counted. The dissector height was set at 16 μm with an upper and lower guard zone of 2 μm. Data was expressed as average ± SEM.

## Additional Information

**How to cite this article**: Garitaonandia, I. *et al*. Neural Stem Cell Tumorigenicity and Biodistribution Assessment for Phase I Clinical Trial in Parkinson’s Disease. *Sci. Rep*. **6**, 34478; doi: 10.1038/srep34478 (2016).

## Supplementary Material

Supplementary Information

## Figures and Tables

**Table 1:** Testing of cGMP grade ISC-hpNSC.

Test	Results
*In Vitro* Assay for Adventitious Viral Contaminants	Negative
*In Vivo* Assay for Adventitious Virus:	Negative
Embryonated Hens Eggs	Negative
Suckling Mice	Negative
Adult Mice	Negative
Guinea Pigs	Negative
Human Epstein-Barr Virus (EBV) DNA by qPCR	Negative
Human Cytomegalovirus (CMV) Virus DNA by qPCR	Negative
Human Parvovirus B19 DNA by q PCR	Negative
Human T-cell Lymphotrophic Virus 1 DNA by qPCR	Negative
Human T-cell Lymphotrophic Virus 2 DNA by qPCR	Negative
Human Herpes Virus 6 (HHV-6) DNA by qPCR	Negative
Human Herpes Virus 7 (HHV-7) DNA by qPCR	Negative
Human Herpes Virus 8 (HHV-8) DNA by qPCR	Negative
Hepatitis B Virus (HBV) DNA by qPCR	Negative
Hepatitis C Virus (HCV) DNA by qPCR	Negative
HIV-1 DNA by qPCR	Negative
HIV-2 DNA by qPCR	Negative
Ultrastructure Cell Morphology and Viral Detection by Thin Section Electron Microscopy	Negative
Polymerase Chain Reaction (PCR)-Based Reverse Transcription Assay (PERT) for Retrovirus Detection	Negative
Mycoplasma (28 day)	Negative
Sterility (14 day)	Negative

**Table 2:** Results of the *in vitro* culture experiment.

Cell Population	Medium	Growth after 1 week	OCT-4 Expression
0.1% hpSC in ISC-hpNSC	E8 medium	Colonies detected	Positive
0.01% hpSC in ISC-hpNSC	E8 medium	Colonies detected	Positive
0.001% hpSC in ISC-hpNSC	E8 medium	No colonies detected	Small signal detected
100% ISC-hpNSC	E8 medium	No colonies detected	Negative
100% hpSC	E8 medium	Confluent in 4 days	Positive
100% hpSC	StemPro NSC medium	Significantly reduced cell numbers	Positive
