# Supplementary material for: Neural Stem Cell Tumorigenicity and Biodistribution Assessment for Phase I Clinical Trial in Parkinson’s Disease
Source: Sci Rep. 2016 Sep 30;6:34478. doi: 10.1038/srep34478 (PMC5055076; doi:10.1038/srep34478)
Supplement: Supplementary Information [file srep34478-s1.pdf]

Neural Stem Cell Tumorigenicity and Biodistribution Assessment for Phase I Clinical Trial in Parkinson's Disease

Ibon Garitaonandia[1*], Rodolfo Gonzalez[1*], Trudy Christiansen-Weber[1], Tatiana Abramihina[1], Maxim Poustovoitov[1], Alexander Noskov[1], Glenn Sherman[1], Andrey Semechkin[1], Evan Snyder[2], Russell Kern[1]

[1]International Stem Cell Corporation, Carlsbad, CA, USA

[2]Sanford-Burnham-Prebys Medical Discovery Institute, La Jolla, CA, USA

*These authors provided equal contribution to this work.

Supplementary Information

Supplementary Methods

Examinations in the acute toxicity study of ISC-hpNSC

General physical examinations were conducted during acclimation which included assessment of the skin, mobility, external orifices, respiration, and reaction to external stimuli. All animals were observed for mortality/moribundity twice-daily except for weekends, where observations were performed at least once-daily. Clinical observations were conducted prior to surgery, two times per day (AM and PM) for three days post-surgery, and then daily for the duration of in-life portion of this study. Assessments included, but were not limited to, observation of activity, posture, respiration, hydration status, stereotypic behavior, surgery site observation, and overall body condition. Individual body weights were recorded on all animals prior to surgery, at least once per week, and on the day of scheduled necropsy (prior to study termination). At 7 days post cell implantation, animals were anesthetized with isoflurane (2.5%) inhalation or by Euthasol. A surgical plane of anesthesia was determined by the lack of paw pinch response. Rats were

transcardially perfused with saline (50 mL) followed by 4% paraformaldehyde (PFA), 200 mL in PBS (pH 7.2–7.4) freshly prepared. Following perfusion, brains were removed and placed in 4% PFA at 4˚C for 24h followed by incubation in 30% sucrose for histological analysis.

Examinations in the tumorigenicity and biodistribution study of ISC-hpNSC

Cageside observations

All animals were observed for morbidity, mortality, injury, and the availability of food and water at least twice daily. On occasion, veterinary consultations were conducted during the course of the study.

Detailed clinical observations

A detailed clinical examination of each animal was performed at receipt, prior to randomization, as necessary during the acclimation period, and weekly during the study. On occasion, clinical observations were recorded at unscheduled intervals. The observations included, but were not limited to, evaluation of the skin, fur, eyes, ears, nose, oral cavity, thorax, abdomen, external genitalia, limbs and feet, respiratory and circulatory effects, autonomic effects such as salivation, and nervous system effects including tremors, convulsions, reactivity to handling, and unusual behavior, and the palpation of masses.

Body weights

Body weights for all animals were measured and recorded at receipt, prior to randomization and surgery, and weekly during the study.

Food consumption

Caged food consumption was measured and recorded weekly during the study.

Clinical pathology

Clinical pathology evaluations were conducted at necropsy at 3, 6, and 9 months. The animals had access to drinking water but were fasted overnight prior to scheduled sample collection. Blood samples (approximately 2.8 to 3 mL) were collected via cardiac puncture or via the vena cava after carbon dioxide inhalation. Samples (approximately 1 to 2 mL) were also collected from animals euthanized in extremis, where possible, and in extremis animals were not fasted prior to blood collection. The samples were collected into tubes containing K_2EDTA for evaluation of hematology parameters and serum separators with no anticoagulant for the clinical chemistry samples. The hematology parameters evaluated were leukocyte count (total and absolute differential), erythrocyte count, hemoglobin, hematocrit, mean corpuscular hemoglobin (volume and concentration), absolute reticulocytes, and platelet count. The clinical chemistry parameters evaluated were alkaline phosphatase, total bilirubin, aspartate aminotransferase, alanine aminotransferase, urea nitrogen, creatinine, total protein, albumin, globulin and albumin/globulin ratio, glucose, total cholesterol, triglycerides, electrolytes (sodium, potassium, chloride), calcium and phosphorus.

Biodistribution analysis

Blood samples (approximately 0.2 to 0.4 mL) were collected at necropsy at 3, 6, and 9 months. The animals had access to drinking water but were fasted overnight prior to scheduled sample collection. Samples (approximately 0.2 to 0.4 mL) were also collected from animals euthanized in extremis, where possible, and in extremis animals were not fasted prior to blood collection. The samples were collected via cardiac puncture or via the vena cava after carbon dioxide inhalation and placed into tubes containing K_2EDTA anticoagulant. Following euthanasia, samples (approximately 100 to 200 mg per organ or approximately 50 to 100 mg for the spleen) were collected from the brain (cerebrum, midbrain, cerebellum, and medulla/pons), adrenal

glands, bone with bone marrow (femur), heart, kidneys (right), liver (left lateral lobe), lung with bronchi, lymph node (cervical), ovaries with oviduct (if applicable), pituitary gland, prostate gland (if applicable), salivary gland (mandibular, parotid, and sublingual), seminal vesicles (if applicable), spinal cord (cervical, thoracic, and lumbar), spleen, testes (right, if applicable), thyroid gland (with parathyroid), and uterus with cervix (if applicable). Tissues were collected from the control animals prior to the treated animals using strict aseptic techniques. One section of each tissue was placed in a labeled 2 mL microcentrifuge tube, snap frozen in liquid nitrogen, and then stored at -60 to -90°C until analyzed. Care was taken to ensure cross contamination between tissues did not occur. Gloves were changed between collection and dissection of each tissue. Non-disposable instruments were wiped down with a 10% bleach solution, rinsed with water, and wiped down with 100% ethanol between groups. Cutting boards were wiped down with a 10% bleach solution, rinsed with water, and wiped down with 100% ethanol between each organ. DNA was extracted from the samples, the concentration determined, and qPCR reactions were performed according to MPI's SOPs.

Histopathology evaluation

Necropsy examinations were performed under procedures approved by a veterinary pathologist on all animals that were euthanized in extremis, found dead, and surviving to the scheduled terminal necropsies. Most animals were euthanized by carbon dioxide inhalation followed by transcardial perfusion with heparinized 0.9% saline. Animals found dead did not undergo saline perfusion. One male was euthanized *in extremis* via carbon dioxide inhalation followed by pneumothorax puncture. The bregma was marked with tissue dye on the brain surface through the skull cap. The animals were examined carefully for external abnormalities including palpable masses. The skin was reflected from a ventral midline incision and any subcutaneous masses

were identified and correlated with antemortem findings. The abdominal, thoracic, and cranial cavities were examined for abnormalities. The organs were removed, examined, and, where required, placed in fixative. All designated tissues were fixed in neutral buffered formalin, except for the testes, which were fixed using a modified Davidson's fixative. Testes were placed into formalin following fixation. Formalin was infused into the lung via the trachea and into the urinary bladder. All tissues were processed to paraffin within 72 hours. When necessary, tissues were stored in 70% ethanol for up to 48 hours prior to placing in paraffin. A full complement of tissues and organs was collected from all animals which included adrenal, blood, bone with marrow (femur), bone marrow smear, brain (cerebrum, midbrain, cerebellum, medulla/pons), gonads (ovary with oviduct or testis), gross lesions, heart, kidney, liver, lung with bronchi, lymph node, pituitary, prostate and seminal vesicle, salivary gland (mandibular/sublingual and parotid), spinal cord (cervical, thoracic, and lumbar), spleen, thyroid/parathyroid, tissues masses and uterus (both horns)/cervix. Microscopic examination of fixed hematoxylin and eosin (H&E)-stained paraffin sections were then performed by a board-certified veterinary pathologist.

Statistical Analysis

The raw data were tabulated within each time interval, and the mean and standard deviation were calculated for each endpoint by sex and group. For each endpoint of the body weights, food consumption, hematology (except leukocyte counts), and clinical chemistry, groups were compared to the vehicle group using group pair-wise comparisons (Levene's/ ANOVA-Dunnett's/ Welch's). Data for the endpoints of leukocyte counts (total and differential), were transformed by either a log transformation prior to conducting the group pair-wise comparisons. The categorical clinical observation data was analyzed by the Cochran Mantel Haenszel test.

For Group Pair-wise Comparisons, endpoints and/or parameters (within each collection interval) that demonstrated variability, and where sample sizes for all groups were three or greater, Levene's test was used to assess homogeneity of group variances. If Levene's test was not significant ($p \geq 0.01$), a pooled estimate of the variance (Mean Square Error or MSE) was computed from a one way analysis of variance (ANOVA) and utilized by a Dunnett's comparison of each treatment group with the control group. If Levene's test was significant ($p<0.01$), comparisons with the control group were made using Welch's t-test with a Bonferroni correction. If the control group had a sample size less than three, no inferential statistics were done. If there was no variability, no inferential statistics were done. In the case that sample size was less than three for at least one treatment group, Levene's method could not be implemented. Groups with sample sizes less than three were excluded from the analysis and control-treatment pair-wise comparisons that satisfied the sample size assumption ($n \geq 3$) were conducted using Welch's t-test with a Bonferroni correction. If there were only two groups involved, the above methodology for testing homogeneity of variance applied and the Dunnett's test reduced to a Student's t-test. All endpoints were analyzed using two-tailed tests.

For endpoints that describe categories rather than numerical measures, a test of association between response and treatment using Cochran Mantel Haenszel tests was conducted. This test was used to assess overall differences among the treatment groups. If the resulting p-value of this test is significant ($p< 0.05$) and there were more than two groups, follow-up comparisons were done on a pair-wise basis, each treatment group to control. All endpoints were analyzed using two-tailed tests.

Supplementary Tables

Supplementary Table S1. Group allocations in acute toxicity study.

Group	Test Article	# Rats and Sex	Injection Sites (# sites)	Concent ration (cells/µl)	Dose/ Site (# cells)	Injection Volume (µl/site)	Total Injection Volume (µl)	Total Dose per Rat (# cells)
1	PBS Control	2M/2F	Striatum (16)<br>SN (2)	0	0<br>0	2 µl<br>2 µl	36 µl	0
2	ISC-hpNSC	2M/3F	Striatum (16)<br>SN (2)	70,750	$1.41x10^5$<br>$1.41x10^5$	2 µl<br>2 µl	36 µl	$2.55x10^6$
3	ISC-hpNSC	3M/2F	Striatum (16)<br>SN (2)	153,000	$3.1x10^5$<br>$3.1x10^5$	2 µl<br>2 µl	36 µl	$5.51x10^6$
4	ISC-hpNSC	3M/2F	Striatum (16)<br>SN (2)	213,500	$4.3x10^5$<br>$4.3x10^5$	2 µl<br>2 µl	36 µl	$7.69x10^6$

Supplementary Table S2. Groups in the tumorigenicity and biodistribution study.

Group	Product	Rat # and Sex	Injection Sites (# sites)	Concent ration (cells/ µl)	Dose/ Site (# cells)	Injection Volume (µl/site)	Total Injection Volume (µl)	Total Dose per Rat (# cells)
1	Control (PBS)	10M/ 10F	Striatum (4)<br>SN (2)	0	0<br>0	2 µl<br>2 µl	12 µl	0
2	Low dose ISC-hpNSC	10M/ 10F	Striatum (4)<br>SN (2)	21,000	$4.2x10^4$<br>$4.2x10^4$	2 µl<br>2 µl	12 µl	$2.5x10^5$
3	Medium dose ISC-hpNSC	10M/ 10F	Striatum (4)<br>SN (2)	42,000	$8.4x10^4$<br>$8.4x10^4$	2 µl<br>2 µl	12 µl	$5x10^5$
4	MFD ISC-hpNSC	10M/ 10F	Striatum (16)<br>SN (2)	214,000	$4.28x10^5$<br>$4.28x10^5$	2 µl<br>2 µl	36 µl	$7.7x10^6$
5	hpSC	10M/ 10F	Striatum (4)<br>SN (2)	42,000	$8.4x10^4$<br>$8.4x10^4$	2 µl<br>2 µl	12 µl	$5x10^5$

Supplementary Table S3. Stereotactic coordinates in acute toxicity study.

**Injection Tracks in Males**	**Target**	**Anterior(+)/ Posterior(-)**	**Left(+)/ Right(-)**	**Dorsal(+)/ Ventral(-)**
1	Striatum	+0.5	+3.0	-4.0, -3.5, -3.0, -2.5
2	Striatum	+0.5	-3.0	-4.0, -3.5, -3.0, -2.5
3	Striatum	-0.7	+3.0	-4.0, -3.5, -3.0, -2.5
4	Striatum	-0.7	-3.0	-4.0, -3.5, -3.0, -2.5
5	Substantia nigra	-5.6	+1.4	-7.5
6	Substantia nigra	-5.6	-1.4	-7.5
**Injection Tracks in Females**	**Target**	**Anterior(+)/ Posterior(-)**	**Left(+)/ Right(-)**	**Dorsal(+)/ Ventral(-)**
1	Striatum	+0.2	+3.0	-3.5, -3.0, -2.5, 2.0
2	Striatum	+0.2	-3.0	-3.5, -3.0, -2.5, 2.0
3	Striatum	-0.7	+3.0	-3.5, -3.0, -2.5, 2.0
4	Striatum	-0.7	-3.0	-3.5, -3.0, -2.5, 2.0
5	Substantia nigra	-5.6	+1.4	-7.0
6	Substantia nigra	-5.6	-1.4	-7.0

Supplementary Table S4. Site coordinates in the tumorigenicity and biodistribution study.

**Injection Tracks in all Males except MFD group**	**Target**	**Anterior(+)/ Posterior(-) (mm)**	**Left(+)/ Right(-) (mm)**	**Dorsal(+)/ Ventral(-) (mm)**
1	Striatum	-0.7	-3.0	-4.0, -3.0
2	Striatum	-0.7	+3.0	-4.0, -3.0
3	Substantia nigra	-5.6	-1.4	-7.5
4	Substantia nigra	-5.6	+1.4	-7.5
**Injection Tracks in MFD Males**	**Target**	**Anterior(+)/ Posterior(-) (mm)**	**Left(+)/ Right(-) (mm)**	**Dorsal(+)/ Ventral(-) (mm)**
1	Striatum	+0.5	-3.0	-4.0, -3.5, -3.0, -2.5
2	Striatum	+0.5	+3.0	-4.0, -3.5, -3.0, -2.5
3	Striatum	-0.7	-3.0	-4.0, -3.5, -3.0, -2.5
4	Striatum	-0.7	+3.0	-4.0, -3.5, -3.0, -2.5
5	Substantia nigra	-5.6	-1.4	-7.5
6	Substantia nigra	-5.6	+1.4	-7.5
**Injection Tracks in all Females except MFD group**	**Target**	**Anterior(+)/ Posterior(-) (mm)**	**Left(+)/ Right(-) (mm)**	**Dorsal(+)/ Ventral(-) (mm)**
1	Striatum	-0.7	-3.0	-3.5, -2.5
2	Striatum	-0.7	+3.0	-3.5, -2.5
3	Substantia nigra	-5.6	-1.4	-7.0
4	Substantia nigra	-5.6	+1.4	-7.0
**Injection Tracks in MFD Females**	**Target**	**Anterior(+)/ Posterior(-) (mm)**	**Left(+)/ Right(-) (mm)**	**Dorsal(+)/ Ventral(-) (mm)**
1	Striatum	+0.2	-3.0	-3.5, -3.0, -2.5, -2.0
2	Striatum	+0.2	+3.0	-3.5, -3.0, -2.5, -2.0
3	Striatum	-0.7	-3.0	-3.5, -3.0, -2.5, -2.0
4	Striatum	-0.7	+3.0	-3.5, -3.0,

				-2.5, -2.0
5	Substantia nigra	-5.6	-1.4	-7.0
6	Substantia nigra	-5.6	+1.4	-7.0

Supplementary Table S5. List of antibodies.

Antigen	Catalog#	Dilution	Application	Manufacturer
OCT-4	53-5841	1:100	Flow Cytometry	eBiosciences
IgG2a	53-4321	1:100	Flow Cytometry	eBiosciences
STEM121	Y40410	1:500	IHC	Takara Bio
IBA-1	P40101-0	1:200	IHC	Pel-Freeze
TH	P60101-0	1:500	IHC	Pel-Freeze
